# High‐Intensity interval training reduces transcriptomic age: A randomized controlled trial

**DOI:** 10.1111/acel.13841

**Published:** 2023-04-20

**Authors:** Trevor Lohman, Gurinder Bains, Steve Cole, Lida Gharibvand, Lee Berk, Everett Lohman

**Affiliations:** ^1^ Loma Linda University School of Allied Health Professions Loma Linda California USA; ^2^ UCLA David Geffen School of Medicine Los Angeles California USA; ^3^ Loma Linda University School of Allied Health Professions, and School of Medicine Loma Linda California USA

**Keywords:** aging, autophagy, biological age, body composition, gene expression, high‐intensity interval training, longevity, transcriptome

## Abstract

While the relationship between exercise and life span is well‐documented, little is known about the effects of specific exercise protocols on modern measures of biological age. Transcriptomic age (TA) predictors provide an opportunity to test the effects of high‐intensity interval training (HIIT) on biological age utilizing whole‐genome expression data. A single‐site, single‐blinded, randomized controlled clinical trial design was utilized. Thirty sedentary participants (aged 40–65) were assigned to either a HIIT group or a no‐exercise control group. After collecting baseline measures, HIIT participants performed three 10 × 1 HIIT sessions per week for 4 weeks. Each session lasted 23 min, and total exercise duration was 276 min over the course of the 1‐month exercise protocol. TA, PSS‐10 score, PSQI score, PHQ‐9 score, and various measures of body composition were all measured at baseline and again following the conclusion of exercise/control protocols. Transcriptomic age reduction of 3.59 years was observed in the exercise group while a 3.29‐years increase was observed in the control group. Also, PHQ‐9, PSQI, BMI, body fat mass, and visceral fat measures were all improved in the exercise group. A hypothesis‐generation gene expression analysis suggested exercise may modify autophagy, mTOR, AMPK, PI3K, neurotrophin signaling, insulin signaling, and other age‐related pathways. A low dose of HIIT can reduce an mRNA‐based measure of biological age in sedentary adults between the ages of 40 and 65 years old. Other changes in gene expression were relatively modest, which may indicate a focal effect of exercise on age‐related biological processes.

## Graphical abstract

This randomized controlled trial finds that one month of high‐intensity interval training can reduce an mRNA‐based measure of biological age in sedentary adults ages 40–65 compared with a no‐exercise control group. Additional hypothesis‐generation bioinformatic analyses suggest that this process might be mediated by changes to autophagy, neurotrophin signaling, and cancer pathways.

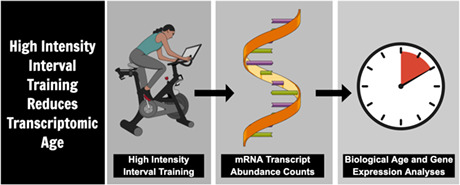

**Abbreviations**
AMPK: Adenosine monophosphate‐activated protein kinase
BFM: Body fat mass
BMI: Body mass index
CDC: Centers for disease control and prevention
cDNA: complementary deoxyribonucleic acid
DEGs: Differentially expressed genes
DNA: Deoxyribonucleic acid
EU: European union
GmbH: Gesellschaft mit beschränkter Haftung
GRCh38: Genome Reference Consortium Human Build 38
h: hour
HIIT: High intensity interval training
HR: Heart rate
ID: Identification
IPAQ: International Physical Activity Questionnaire
IRB: Institutional review board
LLU: Loma Linda University
mRNA: messenger ribonucleic acid
mTOR: mammalian target of rapamycin
mTORC1: mammalian target of rapamycin complex 1
PHQ‐9: Patient health questionnaire 9‐item depression module
PI3K: Phosphoinositide 3‐kinase
PSQI: Pittsburgh sleep quality index
PSS‐10: 10‐item perceived stress scale
RHEB: RAS‐homolog enriched in brain
RNA: Ribonucleic acid
RNAseq: Next generation RNA sequencing
SD: Standard deviation
SMM: Skeletal muscle mass
TA: Transcriptomic age
TAaccel: Transcriptomic age acceleration
TRAP: Transcriptomic age prediction
UCLA: University of California, Los Angeles

## 1 INTRODUCTION

The beneficial effects of exercise on health span and lifespan are among the most well‐documented scientific findings in health science research (Aune et al., 2021; Han et al., 2022; Myers et al., 2002; Northey et al., 2018). Despite this, there are relatively few trials investigating the effects of exercise on gene regulatory mechanisms of health span and life span. Of those that have been performed, most examine the effects of a single bout of exercise on gene expression, rather than repeated bouts (Amar et al., 2021).

Given that many beneficial effects of exercise require repeated bouts over time to manifest, this represents an opportunity for discovery. Consider for example the inappropriate conclusions that could be drawn when studying the effects of a single bout of exercise on muscle hypertrophy, strength, or inflammation. The beneficial effects of exercise on biological aging is likely most apparent when studied over time.

The central theme of molecular biology holds that a cell's function and status are dictated by the specific sets of genes undergoing transcription at any given time, and to what degree these processes are occurring (O'Brien et al., 2012). Genome‐wide expression analyses allow us to take a snapshot of those processes, capturing a gene expression profile at the time of blood draw. A comparison of gene expression profiles before and after an intervention provides the means to identify patterns of differentially expressed genes.

As high‐throughput RNA sequencing becomes more commonplace, gene expression‐based predictive models have emerged. Some of these models are designed to predict biological age (Meyer & Schumacher, 2021; Peters et al., 2015; Ren & Kuan, 2020), or more specifically, transcriptomic age (TA). These models are easily accessible and comprehensive molecular surveys of biological processes that collectively contribute to health span and life span. It is this type of biological age predictor, a “transcriptomic clock” that is used in the trial described here.

The biological age prediction field is diverse and rapidly evolving, with models composed of various inputs (Cesari et al., 2014; Jylhävä et al., 2017; Levine et al., 2018; Lohman et al., 2021; Lu et al., 2019) and predictive capabilities (Li et al., 2020; McCrory et al., 2020). The discrepancy between a participant's actual age and their predicted age is often of particular interest (Fahy et al., 2019; Fiorito et al., 2021). This measure, called age acceleration (biological age minus chronological age), can take a positive or negative value. Positive values are considered hazardous and indicative of an increased aging rate, while negative values are considered beneficial and evidence of a slowed aging rate. Any intervention that reverses age acceleration could therefore be considered beneficial and potentially health protective.

The effect of exercise on various biological age predictors is inconsistent. Most experimental studies that examine the relationship between exercise and biological age use telomere length as their primary biomarker of aging. These results are mixed, with positive relationships, U‐shaped relationships, and no relationship all being reported (Sellami et al., 2021). This could be due to any number of factors, from differences in sample characteristics to the open question of whether telomere length even has utility as a measure of biological age (Glei et al., 2016; Li et al., 2020; Svensson et al., 2014; Vaiserman & Krasnienkov, 2020; Wang et al., 2018).

Fewer studies have been performed using epigenetic alteration, such as DNA methylation or histone methylation/acetylation as an outcome measure. Of those that have been performed, various types of exercise have been shown to induce widespread changes in the methylome and associated gene expression (Barrès et al., 2012; Denham et al., 2015; Nakajima et al., 2010), but the number of studies performed is few.

To the authors' knowledge, only two lifestyle modification trials have utilized a next generation predictor of biological age in humans, such as an epigenetic clock (Fiorito et al., 2021; Fitzgerald et al., 2021), and no prior study has used a transcriptomic predictor of biological age.

The trial described here aims to address this by utilizing high‐throughput RNA sequencing to explore the effects of 12 high‐intensity interval training (HIIT) sessions on biological age as measured by a blood mRNA‐based “transcriptomic clock” (Peters et al., 2015).

To confirm previously observed effects of HIIT on various physiological parameters (Gu et al., 2022; Min et al., 2021; Ouerghi et al., 2017; Su et al., 2019; M. Wewege et al., 2017), changes in body mass index (BMI), body fat mass (BFM), visceral fat area, and measures of psychological stress, depression, and sleep quality were measured.

## 2 METHODS

A single‐blinded randomized controlled trial design was used to investigate the effects of HIIT on the following dependent variables: 10‐item Perceived Stress Scale (PSS‐10) (Lee, 2012), Pittsburgh Sleep Quality Index (PSQI) (Zhang et al., 2020), Patient Health Questionnaire 9‐item depression module (PHQ‐9) (Kroenke et al., 2001; Levis et al., 2020), BMI, BFM, visceral fat area, skeletal muscle mass, waist‐to‐hip ratio, blood pressure, resting heart rate, and whole‐genome RNA expression. The transcriptomic age prediction (TRAP) tool (Peters et al., 2015) was used to assess transcriptomic age and transcriptomic age acceleration (TAaccel = TA – chronological age) using the RNA AGE Calc Shiny App (Ren & Kuan, 2020). The TRAP biological age prediction model was trained to predict chronological age in a meta‐analysis of 14,983 individuals and is based on 11,908 input gene expression levels (Peters et al., 2015).

Trial participants were recruited from local communities surrounding the Loma Linda University campus via flyers, approved social media, and word of mouth. The Loma Linda University Institutional Review Board approved the study on 11/18/2021 (IRB# 5210437, clinicaltrials.gov trial registration ID: NCT05156918). Men and women between the ages of 40 and 65 who self‐identified as nonexercisers were categorized as low activity using the International Physical Activity Questionnaire (IPAQ) (Hagströmer et al., 2006), had no significant change to activity levels within the past 30 days, were not pregnant, had no prior or current history of any condition that would make exercise unsafe, and were not currently taking antibiotics, glucocorticoids, anticoagulants, narcotics, antiepileptics, antipsychotics, or hypoglycemic agents were eligible for participation.

Study participants were instructed to avoid modifying their usual physical activity level or diet for the duration of the four‐week study protocols, except for the additional HIIT assigned to exercise group. All participants maintained a compliance log, comprised of two questions weekly. For the control group: Have you performed more than your usual amount of physical activity this week? Second, have you made any significant changes in your diet this week? For the exercise group: Excluding the exercise assigned to you in this study, have you performed more than your usual amount of physical activity this week? Second, have you made any significant changes in your diet this week?

All participants arrived at the laboratory between the hours of 8 am and 11 am, and baseline measures were obtained. Body composition measurements were obtained using the InBody 770 body composition and body water analyzer (InBody USA), surveys were completed in a private room, and a single vial of blood was collected by a certified phlebotomist from the antecubital vein into a PAXgene® Blood RNA Tube, PLH 16X100 2.5 PLBLCE CLR (Becton Dickinson).

Following the completion of Day‐1 data collection, exercise group participants returned the next day to begin the HIIT protocol that took place at the Loma Linda University Physical Fitness Laboratory. The authors chose a routinely studied 10X1 HIIT protocol that has been determined as safe and effective in various groups, including sedentary individuals (Ito, 2019; Little et al., 2010; Rozenek et al., 2016; M. A. Wewege et al., 2018). The protocol consists of a two‐minute warm‐up and cooldown, with 10, one‐minute high‐intensity exercise intervals at 77%–93% of the participants predicted maximum heart rate (Committee, n.d.) determined using Karvonen's formula (Camarda et al., 2008), followed by one‐minute self‐selected intensity rest periods. The total exercise session lasted 23 min, of which 10 min was high‐intensity exercise and 13 min was warm‐up/rest/cooldown periods.

Participants rotated between three exercise machines (randomly assigned rotation order at outset): A Concept2 rowing ergometer, Concept2 bicycle ergometer, and a Noraxon PhysTread Pressure treadmill. Participants used a different machine each day so that they used each of the three exercise machines once per week.

Each participant wore a Polar Verity Sense Optical HR monitor (Polar Electro, FI) during all exercise sessions, and their real‐time HR was monitored by a lab assistant continuously. The lab assistant would cue the participant to modify effort if HR approached our prespecified target boundaries of 77%–93% predicted HR maximum. This ensured that all participants experienced the same relative workload throughout the exercise intervention.

Following the conclusion of the four‐week control and exercise protocols, all participants returned for result collection. Exercise group data were collected approximately 48 hours after their last HIIT session. All blood collection procedures were standardized, and all collection took place within the same three‐hour window for pre‐ and postblood draws. Samples were stored at −79 degrees Celsius until RNA extraction (Qiagen RNeasy), quality assurance assays, mRNA sequencing, and related statistical analyses of differential gene expression and interpretive bioinformatics were performed by the UCLA Social Genomics Core Laboratory. Transcriptional profiling utilized a high‐efficiency mRNA targeted reverse transcription and cDNA library synthesis system (QuantSeq 3' FWD; Lexogen Inc.) with cDNA libraries sequenced on in Illumina NovaSeq system by Lexogen Services GmbH. Assays targeted 5 million sequencing reads per sample (achieved median = 7.1 million), each of which was mapped to the GRCh38 reference human transcriptome using the STAR aligner (median 99.7% mapping rate) and quantified as gene transcripts per million total mapped reads, with values floored at 1 transcript per million (TPM) to suppress spurious low‐range variability, and log2‐transformed to stabilize variance. One follow‐up sample yielded insufficient sequencing reads for valid analysis (<1 million reads), and that sample and its paired pre‐intervention baseline sample were excluded from all subsequent analyses. These data served as input into the RNA AGE Calc Shiny App for computation of the TRAP RNA age score. RNA AGE Calc Shiny App inputs were as follows: Tissue type: Blood, type of gene expression data: Count, samples used when building the calculator: All samples, gene ID type: Ensembl ID, signature: Peters.

A secondary analysis of differentially expressed genes (DEGs) was performed using two sets of cutoff criteria. First, genes that displayed a group × time interaction expression fold change greater than 1.5 or less than 0.5 were selected for analysis. Also, an exploratory/hypothesis‐generation analysis was performed using more liberal fold change values greater than 1.2 or less than 0.8. Genes were screened into pathway analyses based on differential expression effect size because effect‐size—screened gene lists have been found to be more replicable than those based on *P*‐ or *q*‐value screening (Cole et al., 2003, 2020; Fredrickson et al., 2013; Norris & Kahn, 2006; Shi et al., 2008; Witten & Tibshirani, 2007). Functional enrichment and pathway analyses were performed using Advaita Bio's iPathway Guide (Appendix S2).

### 2.1 Data analysis

Mean ± SD was computed for quantitative variables and frequency (percentage) for categorical variables. Chi‐squared test was used to test the difference between intervention and control group by categorical variables at baseline. Normality of quantitative variables was assessed using Shapiro–Wilk test and box plots. Independent t‐test was used for all continuous and independent variables in both groups at baseline. The Mann–Whitney U test was used to compare the same variables due to small sample and lack of normality on some variables. The dependent paired *t* test was used to compare pre‐ and postvariables in both groups. Also, Wilcoxon Signed‐Rank test was used to compare the pre‐ and postvariables due to small sample and lack of normality on some variables.

Data were analyzed using the SPSS Statistics Software version 28.0 (SPSS Inc.). All analyses were performed at an alpha level of 0.05.

## 3 RESULTS

**FIGURE 1:**
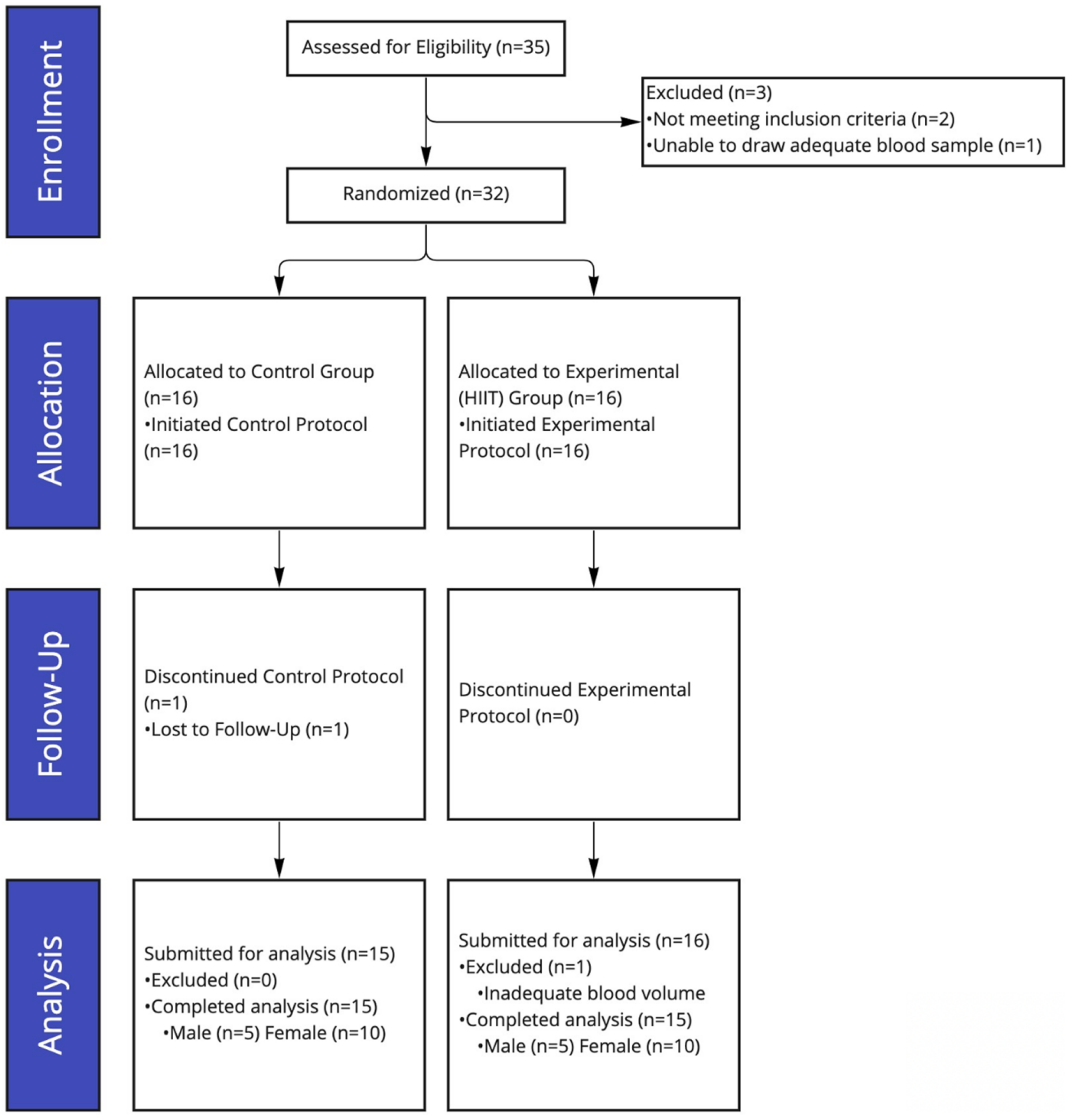
CONSORT chart diagram. Thirty‐five participants recruited, two excluded due to high activity level, one excluded due to an inability to draw blood sample. One control participant lost to follow‐up, one exercise participant excluded from analysis due to low blood volume in postexercise blood sample detected during RNA quality control tests. In total, 15 control participants and 15 experimental participants completed all aspects of the trial and subsequent analysis.

Of the 35 participants screened, 30 subjects satisfied the eligibility criteria, agreed to participate, were randomly assigned to the experimental group (*n* = 15) and the control group (*n* = 15) using computer‐generated block randomization, and completed all subsequent analyses (Figure 1).

Baseline characteristics of participants are shown in Table 1. None of the demographic variables were significant for randomized design.

**TABLE 1:** Selected characteristics of participants at baseline.

Variables	Experimental (*n* = 15)	Control (*n* = 15)	
	**Frequency (%)**	**Frequency (%)**	***p*‐** **value** ^**a**^
Race/Ethnicity			
White	7 (46.67)	5 (33.33)	0.381^a^
Black	2 (13.3)	1 (6.67)	
Hispanic	4 (26.67)	4 (26.67)	
Asian	1 (6.67)	5 (33.33)	
Other	1 (6.67)	0 (0.00)	
Sex			
Female	10 (66.7)	10 (66.70)	0.650^a^
Male	5 (33.3)	5 (33.30)	
Diabetic			
No	13 (86.67)	14 (93.33)	0.414^a^
Yes	0 (0.00)	0 (0.00)	
Prediabetic	2 (13.33)	1 (6.67)	

	Mean ± SD	Mean ± SD	*p*‐value^b^
Age	51.66 ± 7.88	48.57 ± 7.46	0.280
TA (years)	73.40 ± 8.18	67.76 ± 9.32	0.373
TAaccel (years)	21.75 ± 7.60	19.19 ± 7.88	0.089
BMI	31.08 ± 4.93	29.59 ± 5.42	0.436
PHQ‐9	5.33 ± 3.89	6.93 ± 6.87	0.439
PSS‐10	20.13 ± 5.32	21.20 ± 3.36	0.517
PSQI	7.00 ± 3.87	7.60 ± 4.70	0.683
SMM (lbs)	69.47 ± 11.68	63.08 ± 13.96	0.185
BFM (lbs)	74.59 ± 22.13	66.85 ± 22.06	0.389
Visceral fat area cm^2^	162.32 ± 46.18	156.99 ± 58.34	0.783

^a^ Chi‐squared test.

^b^ Independent t‐test or Mann–Whitney U test.

### 3.1 Primary analysis: Transcriptomic age

There were statistically significant differences between groups for TA and TAaccel overtime (*p* = 0.026 and *p* = 0.025, respectively).There was a significant decrease in TA and TAaccel (*p* = 0.043 and *p* = 0.042, respectively) over time for the experimental group, and a nonsignificant increase in TA and TAaccel (*p* = 0.078 and *p* = 0.077, respectively) in the control group (Table 2).

**TABLE 2:** Effects of high‐intensity interval training on transcriptomic age, PHQ‐9, PSS‐10, PSQI, skeletal muscle mass, body fat mass, and visceral fat area. Between‐ and within‐group effects.

Variables	Experimental (*n* = 15)			Control (*n* = 15)			
	Pre	Post	Mean difference (P^a^)	Pre	Post	Mean difference (P^a^)	*p* ^b^
TA (years)	73.40 ± 8.18	69.82 ± 7.69	−3.59 (0.043)	67.76 ± 9.32	71.905 ± 9.32	3.29 (0.078)	0.026
TAaccel (years)	21.75 ± 7.60	17.91 ± 9.19	−3.84 (0.042)	19.19 ± 7.88	22.40 ± 6.82	3.21 (0.077)	0.025
PHQ‐9	5.33 ± 3.89	2.27 ± 1.91	−3.07 (0.001)	6.93 ± 6.89	7.00 ± 5.88	0.07 (0.482)	0.063
PSS‐10	20.13 ± 5.32	19.80 ± 3.99	−0.33 (0.265)	21.20 ± 3.36	19.73 ± 5.18	−1.47 (0.027)	0.739
PSQI	7.00 ± 3.87	5.47 ± 3.54	−1.53 (0.021)	7.60 ± 4.70	7.67 ± 4.39	0.07 (0.335)	0.158
SMM (lbs)	69.47 ± 11.68	69.62 ± 11.53	0.15 (0.338)	63.08 ± 13.96	63.47 ± 13.43	0.39 (0.228)	0.705
BFM (lbs)	74.59 ± 22.13	73.12 ± 22.23	−1.47 (0.015)	66.85 ± 22.06	67.01 ± 22.72	0.17 (.122^b^)	0.263
BMI (kg/m^2^)	31.08 ± 4.93	30.85 ± 4.95	−0.23 (0.024)	29.59 ± 5.42	29.51 ± 5.10	−0.08 (0.299)	0.513
Visceral Fat Area cm^2^	162.32 ± 46.18	158.07 ± 46.06	−4.25 (0.008)	156.99 ± 58.34	154.33 ± 58.34	−2.66 (0.031)	0.426

*Note*: Values are presented as mean ± SD.

Abbreviations: BFM, Body Fat Mass (lbs.); BMI, Body Mass Index; PHQ‐9, Patient Health Questionnaire 9‐item depression module; PSQI, Pittsburgh Sleep Quality Index; PSS‐10, 10‐item Perceived Stress Scale; SMM, Skeletal Muscle Mass (lbs.); TA, transcriptomic age; TAaccel, Transcriptomic Age Acceleration (transcriptomic age minus chronological age).

^a^ *p*‐values for the null hypothesis that mean post value is increased from mean pre value (one‐tailed test).

^b^ *p*‐values for the null hypothesis that there is no difference between groups (Group × Time interaction).

### 3.2 Secondary analyses: Gene expression analyses, body composition, depression, sleep, and stress ratings

There were no statistically significant differences between groups for stress, sleep, or depression. There was a significant decrease in mean PHQ‐9 (depression) and PSQI (sleep) (*p* = 0.001 and *p* = 0.021, respectively), over time within the experimental group and a nonsignificant increase in the control group (*p* = 0.482 and *p* = 0.335, respectively) (Table 2). Also, there was a small, nonsignificant increase in mean skeletal muscle mass (SMM) for the experimental and control group (*p* = 0.338 and *p* = 0.228, respectively). Lastly, there was a nonsignificant decrease in mean PSS‐10 for the experimental group but a significant decrease in mean PSS‐10 for the control group (*p* = 0.265 and *p* = 0.027, respectively).

There was a significant decrease in BFM, BMI, and visceral fat area (*p* = 0.015, 0.024, and 0.008, respectively) over time within the experimental group, a nonsignificant increase in BFM (*p* = 0.122), a nonsignificant decrease in BMI (*p* = 0.299), and a significant decrease in visceral fat area (0.031) in the control group (Table 2). However, there was no significant difference between groups for BFM, BMI, and visceral fat area (*p* > 0.05 for all) (Table 2).

The group × time interaction gene expression analysis identified 98 genes that were differentially expressed using routinely accepted fold change cutoff values (86 upregulated genes >1.5‐fold change, and 12 downregulated genes <0.5‐fold change in the exercise group compared with control group). This number is insufficient for secondary enrichment analyses. Using more liberal fold change values of >1.2 and <0.8 for this exploratory analysis, 2653 DEGs were identified (1075 upregulated genes >1.2‐fold change, and 1778 downregulated genes <0.8‐fold change) (Appendix S1). In addition, 1365 Gene Ontology (GO) terms, 477 gene upstream regulators, 231 chemical upstream regulators, and 259 diseases were found to be significantly enriched before correction for multiple comparisons (Appendix S2).

Pathway analysis was performed using Advaita Bio's iPathwayGuide, which scores pathways using the Impact Analysis method (Draghici et al., 2007; Tarca et al., 2009). Impact analysis uses two types of evidence: (i) the overrepresentation of differentially expressed (DE) genes in a pathway and (ii) the perturbation of that pathway computed by propagating the measured expression changes across the pathway topology. The top five pathways identified by this analysis and their associated P‐values are as follows: Human T‐cell leukemia virus 1 infection (*p*‐value = 2.033e‐7, *p*‐value (FDR) = 3.888e‐5, *p*‐value (Bonferroni) = 6.851e‐5), pathways in cancer (*p*‐value = 2.308e‐7, *p*‐value (FDR) = 3.888e‐5, *p*‐value (Bonferroni) = 7.776e‐5), neurotrophin signaling pathway (*p*‐value = 4.670e‐7, *p*‐value (FDR) = 5.246e‐5, *p*‐value (Bonferroni) = 1.574e‐4), RNA degradation (*p*‐value = 1.140e‐6, *p*‐value (FDR) = 5.939e‐5, *p*‐value (Bonferroni) = 3.842e‐4), and autophagy (*p*‐value = 1.190e‐6, *p*‐value (FDR) = 5.939e‐5, *p*‐value (Bonferroni) = 4.009e‐4). A detailed description of these results, including pathway diagrams, is shown in Appendix S2.

## 4 DISCUSSION

In this randomized controlled trial examining the effects of HIIT on an RNA‐based measure of biological age, participants in the HIIT group showed significant reductions in TA and TAaccel. This improvement in biological age coincided with improvements to body composition, ratings of sleep quality, and ratings of depression within the exercise group. These results suggest that exercise exerts an effect on age‐related patterns of gene expression and that such effects could potentially contribute to the positive health and longevity effects associated with exercise.

### 4.1 Transcriptomic age and transcriptomic age acceleration

Both groups began the trial with positive transcriptomic age acceleration. In other words, mean transcriptomic age was higher than mean chronological age in both groups at baseline. In the exercise group, TA and TAaccel decreased following the HIIT protocol, while both measures increased in the control group over the same time frame. Although the observed changes in both groups were relatively large considering the trial's length, changes in TA and TAaccel only achieved statistical significance within the exercise group. Specifically, a 3.59‐year reversal of TA was observed in the exercise group, which can be interpreted as the average gene expression pattern among exercise participants changing to reflect that of a person 3.59 years younger than their mean baseline TA.

Interestingly, changes in TA and TAaccel were the only statistically significant Group X Time observations among all measured variables. This may suggest a focal effect of HIIT on biological aging processes; however, it is important to acknowledge that the correlation between changes in TA and long‐term aging processes is unknown. While this metric is shown to be associated with chronological age, transcriptional processes are rapidly modifiable and the durability of short‐term changes in TA over time is not clear.

This trial supports our prespecified hypothesis that HIIT was capable of beneficially modulating transcriptomic age, but future research should obtain TA measurements over longer time frames, utilize a larger sample size, include multiple TA measurement intervals, and include longitudinal follow up to establish the durability of TA change over time and to ascertain its correlation with aging and disease processes. Future studies may additionally include numerous biological age prediction models to establish convergent validity across different methodologies.

The TRAP model consistently overestimated participant age in all blood samples. The authors believe this is due to differences in data type between the TRAP training dataset and our sample. The TRAP model was developed and trained using microarray data (Peters et al., 2015), while our transcript counts were derived from RNAseq data. However, since this applies equally to all blood samples regardless of group assignment or time of collection, there is no reason to believe that this introduced any bias into the observed magnitude and direction of TA change.

### 4.2 Gene expression

The use of a gene expression‐based measure of biological age has the added advantage of facilitating additional transcriptomic analyses, which could shed light on the mechanisms underlying exercise's effect on aging processes. However, in an untargeted genome‐wide expression analysis, 12 HIIT sessions had only modest effects on gene expression.

Although there were transcriptomic effects associated with HIIT, less than 100 genes displayed a fold change greater than 1.5 or less than 0.5, the values typically used to identify DEGs. This DEG count is less than the amount required for subsequent higher‐order bioinformatic analyses such as a functional enrichment analysis.

While these modest findings may seem surprising given the systemic physiological changes induced by exercise, it is important to remember that this trial examined the effects of a 1‐month HIIT protocol on steady‐state expression levels. The follow‐up blood draw occurred approximately 48 h after the final exercise session, meaning that whole‐genome expression was assessed while the participants were not experiencing the acute physiological aftereffects of exercise. Given the small dose and duration of our exercise protocol and the small sample size, this modest between group effect may not be surprising.

The 48‐h delay in follow‐up blood collection was specified to minimize changes in inflammatory processes and cell composition following the last exercise bout. This helped isolate a more accurate picture of “per cell” transcriptional changes rather than transitory changes in cell composition. This is necessary because acute exercise modifies the presence of all major leukocyte populations in peripheral blood as a result of sympathetic stimulation, shear stress, inflammatory signaling, and other processes (Peake et al., 2016). Most of these changes to circulating white blood cell profile are returned to baseline within hours of exercise cessation (Walsh et al., 2011). Some changes, however, such as depression of specific lymphocyte populations or neutrophil elevation can persist longer. The evidence suggests that a return to baseline can be expected within 4–6 h for lymphocyte subpopulations and within 24 h for neutrophilia (Walsh et al., 2011). The role of cell composition can never be fully accounted for without flow cytometry, and future researchers should consider employing this mechanism to fully control for this potential confounder. The authors' aim was to assess changes in basal aging physiology, and the 48‐hour gap between the final exercise session and blood draw helped ensure this.

An exploratory genome‐wide discovery analysis using more liberal fold change cutoff values (greater than 1.2 or less than 0.8) revealed 1075 upregulated transcripts and 1778 downregulated transcripts potentially associated with HIIT (Appendix S1). The subsequent bioinformatic analyses associated with these DEGs were performed using Advaita Bio's iPathwayGuide. This analysis suggests that autophagy processes, cancer pathways, neurotrophin signaling pathways, mRNA degradation processes, and other pathways were modified by HIIT (Appendix S2). These modifications are particularly interesting in the context of aging, especially autophagy. Various age‐related signaling pathways were modified including mTOR signaling, AMPK signaling, PI3K signaling, and insulin signaling pathways. Inhibition of three of five mTORC1 complex component genes (Raptor, Deptor, and mTOR) was noteworthy, since mTORC1 inhibition is associated with increased life span in every species studied so far, including humans (Papadopoli et al., 2019; Weichhart, 2018). An upstream mTORC1 activator, RHEB, was also inhibited. RHEB activates mTOR by antagonizing its endogenous inhibitor, FKBP38 (Bai et al., 2007). HIIT‐induced RHEB inhibition could therefore lead to mTORC1 inhibition. Given the exploratory nature of these enrichment analyses, and the relatively liberal threshold for DEG detection, however, these results should be treated as descriptive hypotheses to be tested in future research using more rigorous methods.

The raw transcript count per genome feature for all samples is available in Appendix S3. Normalized data (log2‐transformed transcripts per million mapped reads for each sample, with values floored at log2‐0 = transcript per million to suppress spurious variability for low count values) are available in Appendix S4.

### 4.3 Body composition and Self‐Reported measures of sleep quality, stress, and depression

Greater improvements to body composition were observed in the exercise group. Previous work suggests that the effects of exercise on biological age are mediated by changes in body composition (Kresovich et al., 2021). This seems to support our findings, as reduced TA coincided with improvements to BMI, body fat mass, and visceral fat area in the exercise group. Improvements in depression ratings and sleep quality (PHQ‐9 and PSQI score, respectively) were also seen in the exercise group over time.

Observed changes in body composition were consistent with previous studies' findings, indicating that this study's specific implementation of HIIT imparted the expected effects demonstrated in prior investigations. This serves as a positive control, or paradigm validation of the trial's specific HIIT intervention.

### 4.4 Significance

Starting and adhering to a new exercise program is difficult, a fact perhaps best illustrated by the current sedentary behavior rate in the United States. A recent Centers for Disease Control and Prevention (CDC) telephone survey estimates that more than 25% of Americans participate in no physical activity outside of work (CDC, 2022) and contrary to popular opinion, this is not a uniquely American problem. A large European Union study found that 53.1% of the adult EU population participated in >4.5 h of sedentary behavior per day (López‐Valenciano et al., 2020).

High‐intensity interval training is a potential tool to help combat this trend given the decreased time commitment (Cobbold, 2018; Ito, 2019) and similar (or improved) health benefits to those bestowed by other forms of exercise (Hannan et al., 2018; Scott et al., 2019), but with increased adherence and compliance rates (Ito, 2019).

Despite the modest gene expression findings generally, the prespecified hypothesis regarding HIIT‐induced transcriptomic age reversal was proven out by the analysis. Considering that each exercise participant completed a combined 276 min of exercise over 1 month, only 2 h of which was high‐intensity exercise, the effect of HIIT on biological age appears promising.

This study further supports the notion that adding even a small amount of exercise can be beneficial, given that just 12 HIIT sessions were shown to significantly improve TA and TAaccel. To the authors' knowledge, this is the first trial to demonstrate the effects of a specific exercise protocol on a next‐generation measure of biological age. The results suggest that exercise may exert a focal effect on age‐related patterns of gene expression and that such effects could potentially contribute to the positive health and longevity effects associated with exercise.

## 5 CONCLUSION

A low dose of HIIT is sufficient to reduce transcriptomic age in sedentary, middle‐aged adults. Other changes in gene expression were relatively modest in comparison with the transcriptomic age reduction effect size. These findings, along with modification to autophagic pathways, may indicate a particular HIIT specificity for age‐related biological pathway modulation. The key observations presented here, namely reduced transcriptomic age, indicate that exercise may potentially improve health and longevity by altering age‐related transcriptional processes.

## AUTHOR CONTRIBUTIONS

T.L. conceived and designed the study, performed transcriptomic age and associated enrichment analyses. G.B. coordinated the project and together with T.L. and E.L. designed the study. S.C. performed all RNA extraction, quality control, and associated transcriptomic analyses. L.G. performed associated statistical analyses. All authors contributed to and approved the manuscript.

## FUNDING STATEMENT

Research funded by School of Allied Health Professions, Loma Linda University.

## CONFLICT OF INTEREST STATEMENT

The authors declare no competing interests.

## Supporting information


**Appendix S1:**HIIT Time X Intervention Differentially Expressed Gene List

**Appendix S2:**iPathwayGuide Pathway Analyses

**Appendix S3:**Raw Transcript Count per Genome Feature

**Appendix S4:**Normalized Expression Data

## Data Availability

The data that support the findings of this study are available from the corresponding author, [T.L.], upon reasonable request.
